# Immunotherapy targeting esophageal cancer stem cells

**DOI:** 10.1186/2051-1426-1-S1-P178

**Published:** 2013-11-07

**Authors:** Dongli Yue, Hongzheng Ren, Lan Huang, Yi Zhang

**Affiliations:** 1 The Biotherapy Center, The First Affiliated Hospital of Zhengzhou University, Zhengzhou, China; 2 The Department of Oncology, The First Affiliated Hospital of Zhengzhou University, Zhengzhou, China; 3 The Department of Oncology, The Center Hospital of Kaifeng, Henan University, Kaifeng, China

## 

Esophageal cancer is one of most common malignancies. Cancer stem cells (CSC) are considered to be resistant to chemotherapy and radiotherapy, which might be associated with the recurrence of the tumor after treatment. To find the therapeutic procedures targeting CSCs, the different methods have been used to identify the esophageal CSC-related genes and surface markers. The esophageal CSCs were found to resist the chemotherapy and the mechanism was explored as well. We also found the levels of tumor associated antigens in esophageal CSCs have differences comparing with the non-stem cancer cells. To understand if the tumor associated antigens can be used as targets by immune cells, we cloned the tumor antigen specific T cells and analyzed the T cell recognition of esophageal CSC both in vitro and in vivo. We found that the immunotherapy might be one of the optimal procedures targeting both non-stem cancer cells and esophageal CSC.

